# The effects of chronic stress on health: new insights into the molecular mechanisms of brain–body communication

**DOI:** 10.4155/fso.15.21

**Published:** 2015-11-01

**Authors:** Agnese Mariotti

**Affiliations:** 1 Science Writer in Lausanne, Switzerland

**Keywords:** atherosclerosis, brain, chronic stress, depression, immune system

**Figure 1.**
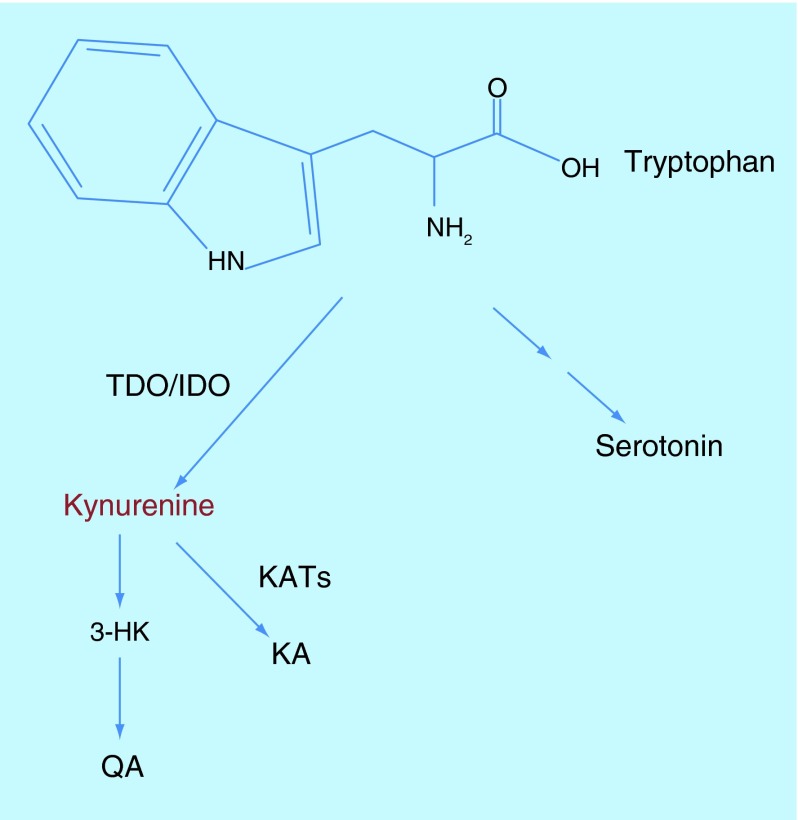
**The Kynurenine pathway of tryptophan degradation.**

**Figure 2.**
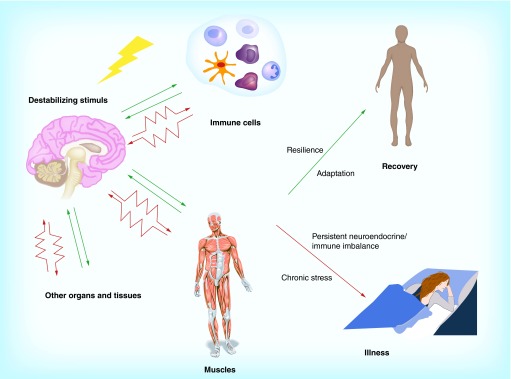
**The effects of psychological stress on brain periphery networks.**

Today's life rhythms and demands are often challenging and require intense physical and psychological efforts in order to be sustained. An individual reacts to physical and mental strain that is potentially health threatening by activating interconnected neuroendocrine circuits. This response allows the body to face and deal with the challenge and re-establish homeostatic equilibrium. If the individual perceives a noxious stimulus as too intense, or its duration as too long, he may fail coping with it, and incur maladaptation. In this case, the stress response does not resolve into a state of balance (either similar or new, i.e., adapted, compared with the state before stress hits), neuroendocrine parameters remain altered, and illness may ensue.

It is clear that stress has both a physical (objective) and a psychological (subjective) component: the latter, as described by Koolhaas and colleagues, depends on the individual perception of its predictability and controllability [1]. The way a person can anticipate a certain stressor and then control it, largely defines the resulting stress response, how promptly and efficiently it is activated promoting adaptation, and how fast it is turned off once equilibrium has been recovered.

The time course of the stress response, characterized by measurable neuroendocrine and behavioral indexes, thus reveals whether a destabilizing stimulus is manageable, or conversely, cannot be handled and consequently becomes harmful.

This implies that not all stimuli that elicit strong neuroendocrine responses are real stressors, but only those that exceed the individual's ability to change and adapt.

Cortical centers in the brain sense a disturbing stimulus and respond by activating pathways that through the limbic system stimulate peripheral networks, including the sympathetic–adrenal–medullary axis and the renin-angiotensin system, and later the hypothalamic–pituitary–adrenal (HPA) axis [2]. A cascade of events follows that results in the orchestration of a complex response. Adrenaline and other hormones, and neuropeptides are produced and regulate cardiovascular and metabolic functions (inducing, for instance, increases in heart rate, breath frequency, glucose release) for a prompt response concerted to overcome the challenge.

If the distressing stimulus persists, the HPA axis kicks in to sustain the immediate reaction mediated by the centrally activated peripheral systems. The HPA response starts with the hypothalamus delivering corticotropin-releasing hormone to the pituitary and culminates with the stimulation of the adrenal cortex by the pituitary-derived adrenocorticotropic hormone to produce glucocorticoids (GCs). Most organs and tissues, including sympathetic nerves, immune cells and several brain regions express GC receptors and are responsive to GCs induced by stress. Consequently, these hormones participate in the regulation of disparate stress-associated processes, from the modulation of cardiovascular effects and the immune function, to the eventual dampening of the stress response through inhibition of the HPA axis when adaptation is attained.

In situations in which the stressor is overwhelming and cannot be resolved, stress becomes chronic. In this case, the GC-dependent negative feedback mechanism that controls the stress response does not work, GC receptor resistance develops, and the systemic levels of the molecular mediators of stress remain high, compromising the immune system and damaging in the long-term multiple organs and tissues [3].

## What is the actual impact of chronic stress on health?

When considering the numerous cellular targets of the chemical mediators of stress, one would expect that protracted, stress-dependent neuroendocrine dysregulation may damage directly or through functional circuits practically all organs and tissues. To clarify this assumption and identify the biochemical pathways significantly impaired by chronic stress to the extent of producing illness, researchers have on one hand searched for putative morphological tissue alterations associated with stress, and on the other analyzed the molecular mechanisms of action of the main stress hormones.

### Effects of chronic stress on brain structure

It has been shown that chronic stress is linked to macroscopic changes in certain brain areas, consisting of volume variations and physical modifications of neuronal networks. For example, several studies in animals have described stress-related effects in the prefrontal cortex (PFC) and limbic system, characterized by volume reductions of some structures, and changes in neuronal plasticity due to dendritic atrophy and decreased spine density [4]. These morphological alterations are similar to those found in the brains of depressed patients examined postmortem, suggesting that they could also be at the basis of the depressive disorders that are often associated with chronic stress in humans. This hypothesis is supported by imaging studies that evidenced structural changes in the brain of individuals suffering from various types of stress-related disorders, such as those linked to severe traumas, major negative life events or chronic psychosocial strain. In particular, Blix and colleagues observed atrophy of the basal ganglia and significantly reduced gray matter in certain areas of the PFC in subjects afflicted with long-term occupational stress [5]. In general, the consequences of these alterations in a brain region can expand to other functionally connected areas, and potentially cause those cognitive, emotional and behavioral dysfunctions that are commonly associated with chronic stress, and that may increase vulnerability to psychiatric disorders.

### Interlink between the brain & the immune system

The understanding of the molecular circuits that underlie brain architectural changes and medical conditions linked to chronic stress is just at the beginning. Research in this area has centered primarily on the signaling functions of those molecules that are directly induced by stress through the activation of the sympathetic-adrenal-medullary and HPA networks, focusing on their possible cellular targets. Since receptors for stress neuropeptides and hormones are broadly expressed in immune cells [6], most studies have concentrated on the effects of stress on the immune system (IS). In fact, psychological stress can induce the acute phase response commonly associated with infections and tissue damage, and increase the levels of circulating cytokines and of various biomarkers of inflammation [2]. As suggested by Maier and Watkins, the interlink between the stress response and inflammation elicited by the IS can be explained from the evolutionary perspective by considering that the stress response is an adaptive process developed by co-opting the IS mechanisms of defense [7]. In this frame, a psychological stressor, perceived by the brain as ‘danger’, that is, potentially harming, sets in motion a neuroimmune circuit that stimulates the IS to mount a protective reaction intended to prevent damage, repair it and restore homeostasis.

This neuroimmune communication is bidirectional because the cytokines produced by stress-stimulated immune cells also convey a feedback to the nervous system, further modulating the release of stress hormones in the brain, as well as brain activity that regulates behavior and cognitive functions. In a situation of chronic stress, the neuroimmune axis can be overstimulated and breaks down, thus causing neuroendocrine/immune imbalances that establish a state of chronic low-grade inflammation, a possible prelude to various illnesses [8].

Diseases whose development has been linked to both stress and inflammation include cardiovascular dysfunctions, diabetes, cancer, autoimmune syndromes and mental illnesses such as depression and anxiety disorders.

Persistent, abnormal levels of cytokines and stress chemical mediators in the brain may also damage the parenchyma and cause neuronal death, thus contributing to the brain structural changes associated with chronic stress that are described above [9].

Despite the large number of studies that have addressed the biological effects of chronic stress and their impact on human health, the emerging picture still merely outlines the biochemical and functional responses of the nervous and immune systems to long-term stress, highlighting some nodes of information exchange between the two networks, but still lacking essential elements concerning additional cellular players, and functional and molecular mechanisms.

Some recent studies have, however, significantly improved our knowledge of how chronic stress promotes two of the diseases that have long been associated with it: atherosclerosis and depression.

### Effects of chronic stress on hematopoietic stem cells in cardiovascular diseases

Heidt and colleagues demonstrated how stress increases the levels of circulating inflammatory leukocytes by direct stimulation of hematopoietic stem cell proliferation [10]. In this new pathway, stress induces the release of noradrenaline by sympathetic nerve fibers targeting blood vessels in the bone marrow of mice. The catecholamine then acts on mesenchymal stem cells located in the hematopoietic niche, which express high levels of the β3 adrenergic receptors. One of the consequences of this interaction is the downregulation of the chemokine CXCL12, a known target of noradrenaline, which is normally produced by several types of niche cells, including mesenchymal stem cells. This releases the inhibition typically exerted by CXCL12 on the proliferation of hematopoietic stem and progenitor cells and on leukocyte migration, thus promoting cell division and leukocyte mobilization into the bloodstream.

Predictably, the inflammatory response induced by the activation of this neuroimmune pathway may have adverse health effects, in particular by exacerbating pre-existing medical conditions. Specifically, the study demonstrated that this mechanism became activated when atherosclerosis-prone mice ApoE^-/-^ were subjected to long-term stress, leading to enhanced recruitment of inflammatory cells in atherosclerotic plaques, higher levels of proteases and increased plaque fragility. Most interestingly, β3-adrenergic receptor blockers opposed leukocyte production and mobilization, thus counteracting plaque inflammation.

When shifting their attention to human subjects, the scientists determined that individuals under considerable occupational stress had significantly more circulating leukocytes compared with when they were not working, suggesting that the neuroimmune mechanism they discovered might be set off by chronic stress and sustain an inflammatory reaction also in humans. If demonstrated, this would open the way to conceptually new therapeutic possibilities not only for atherosclerosis and its related complications, but also for other stress-related diseases that are aggravated by chronic inflammation.

### Mechanisms of chronic stress-associated depression & brain–skeletal muscle communication

The mechanisms by which stress chemicals may induce depression are mostly undetermined. Research in this field is starting to define how multiple, independent, though often interconnected biochemical pathways affected by chronic stress concur to promote this disease.

Ota and colleagues have identified a molecular mechanism triggered by chronic stress that contributes to neuronal atrophy in specific brain areas, an anomaly that is typically observed in depressed patients, independent of the cause of depression [11]. In their study, they observed that persistent high levels of GCs, resulting from stress-induced hyperactivation of the HPA axis, stimulate the production of the molecule REDD1 in the PFC of rodents subjected to prolonged stress. REDD1 is generally induced by a variety of stressors – from energy stress, to hypoxia, to DNA damage – in most tissues and inhibits the kinase mTORC1, thus altering the phosphorylation state and function of its targets. In the brain, the interference of REDD1 with mTORC1 signaling ultimately impinges on neuronal protein synthesis, spine formation and synaptic plasticity. The inhibition of mTORC is pivotal for synaptic impairment and appears to be a central endpoint of molecular pathways turned on by chronic stress. In fact, also the decrease of brain-derived neurotrophic factor levels in response to chronic stress disrupts mTORC1 function.

Proinflammatory cytokines induced by stress are also involved in the development of chronic stress-associated depression [12]. The acute phase response generally triggered by a harmful factor implies the so-called sickness behavior that includes symptoms similar to those typical of depressive disorders, like social withdrawal, decreased physical activity, fatigue, somnolence, mood and cognitive alterations. This adaptive response is orchestrated by cytokines, and is meant to divert an individual from normal activities in order to save energy, thus facilitating a reaction against the challenge, and subsequent recovery. In the case of chronic inflammation that may set in with prolonged stress, persisting cytokine signaling in the brain prevents the resolution of sickness behavior that consequently can degenerate into depression. The biochemical mechanisms underlying cytokine-induced depression are not well defined, but they may involve alterations of serotonin and glutamatergic transmission, and induction of GC resistance [12].

One of the pathways that are implicated drives the oxidation of tryptophan (a precursor of serotonin) to Kynurenin (Kyn) and results in the brain production of several neuroactive molecules (Figure 1) [12]. The enzymes tryptophan 2,3-dioxygenase and indoleamine 2,3-dioxygenase trigger the Kyn pathway in the liver and extrahepatically respectively, and can both be activated by chronic stress: tryptophan 2,3-dioxygenase in fact responds to GCs, while indoleamine 2,3-dioxygenase is induced by proinflammatory cytokines. The Kyn pathway not only affects the brain levels of serotonin and thus serotonergic transmission and its mood and behavioral effects, but it is also responsible for the production of tryptophan metabolites that have neuroinflammatory properties or can affect glutamatergic neurotransmission in the brain either positively or negatively. It follows that a shift in the pathway that favors the production of 3-hydroxykynurenine – an inducer of reactive oxygen species and inflammation – and quinolinic acid – an *N-*methyl-d-aspartate receptor agonist – over the antioxidant and *N-*methyl-d-aspartate receptor inhibitor kynurenic acid, may promote depression [12].

There is evidence that physical exercise can sustain brain health by regulating the production of neurotrophic factors, neurotransmitters, as well as inflammatory molecules. This can translate in a general enhancement of cognitive abilities, a reduced risk of neurodegenerative diseases and a mitigation of depression [13].

An interesting study has recently demonstrated that a key mechanism by which physical exercise counteracts chronic stress-dependent depression is the modulation of the Kyn pathway of tryptophan degradation [14]. In this work, Agudelo and colleagues demonstrated that skeletal muscle contraction during protracted training induces locally the interaction of the transcriptional coactivator PGC-1α1 with the transcription factors PPARα/δ, thus increasing in the muscles the expression and activity of a set of kynurenin aminotransferases (KAT). KATs catalyze the peripheral transformation of tryptophan-derived Kyn into kynurenic acid, causing a drop in the levels of circulating Kyn. Since Kyn can cross the blood–brain barrier, its peripheral catabolism has the effect to reduce also its brain concentration and so the production of neurotoxic molecules along the 3-hydroxykynurenine and quinolinic acid branch of the degradation pathway. In mice, under conditions that mimic chronic stress, the overexpression in skeletal muscles of PGC-1α1 prevents neuroinflammation, synapses impairment and depression-like behaviors that are instead observed in control animals. Similarly, the scientists found that exercise training stimulates the PGC-1α1-KAT pathway also in humans, and through this mechanism, potentially regulates those Kyn-dependent toxic effects in the brain that contribute to chronic stress-associated depression.

By identifying new signaling cascades implicated in the regulation of depression caused by chronic stress, Ota's and Agudelo's studies suggest novel areas of investigation for therapy development. Drugs that inhibit REDD1 effects on mTORC1, or that modulate brain levels of Kyn by enhancing its peripheral metabolism could improve the treatment of depression maybe also when it is not stress related. Since depressed individuals are usually reluctant to carry out regular physical exercise, drugs that mimic exercise training and reduce the plasma concentration of Kyn by acting peripherally would be of particular interest.

### Biological & social implications of the latest findings in chronic stress research

These studies significantly improve our understanding of the interactions between the nervous systems and peripheral tissues and organs, and how their alterations can cause illness (Figure 2). An important discovery is that if on one hand chronic stress can cause immune dysfunctions, that is, impair a peripheral function, on the other hand proper stimulation of a peripheral tissue like skeletal muscles can relieve stress symptoms and protect the brain, possibly favoring recovery. This suggests that programs of physical exercise should be formally proposed as a preventive measure to people known to be exposed to intense stress (eg., work-related stress), and could be prescribed as a form of therapy in combination with other treatments to ease mood and cognitive deficits caused by chronic stress.

In addition, Heidt's study suggests another intriguing possibility: if chronic stress can stimulate hematopoietic stem and progenitor cells through the activation of the peripheral nervous system, it is plausible that it can activate also other types of stem cells in other tissues by a similar mechanism. Bidirectional communication could in principle exist between the nervous system and every organ and tissue, and represents a general mechanism of nervous control of tissue homeostasis. A few recent studies have provided evidence that supports this hypothesis [15–16]. One of the resulting and provoking implications is that chronic stress could promote cancer development also by direct induction of uncontrolled cell proliferation.

The recognition that chronic stress can cause serious diseases has intensified research to determine the biochemical perturbations that compromise homeostasis to a degree that prevents spontaneous recovery. The picture is very complex because chronic stress appears to affect organ and system functions at multiple levels. Yet, it is by pinpointing specific biochemical processes affected by chronic stress that it will be possible to envisage solutions to stimulate resilience and control stress-dependent diseases.

It is clear that in the case of illnesses caused by heightened occupational stress, priority should be given to preventive interventions with the purpose of creating and maintaining work conditions respectful of human physiological, emotional and social needs: in other words, the work environment should stimulate growth and productivity while supporting each individual in their challenges. If certain measures could be implemented through official regulations that assure, for example, fair contracts, training, and sensible work schedules in relation to the type and load of responsibilities and the levels of physical and mental engagement implied by the job, others are less manageable because they strictly depend on human factors. Elements like discordant interactions with coworkers and superiors’ demands beyond formal agreements, that are quite common in very competitive work environments, can sharpen tensions and exaggerate the psychosocial strain to the point of causing illness, yet they usually remain overlooked and uncontrolled [17].
